# Predicting forest insect flight activity: A Bayesian network approach

**DOI:** 10.1371/journal.pone.0183464

**Published:** 2017-09-27

**Authors:** Stephen M. Pawson, Bruce G. Marcot, Owen G. Woodberry

**Affiliations:** 1 Scion, Riccarton, Christchurch, New Zealand; 2 US Forest Service, Pacific Northwest Research Station, Portland, Oregon, United States; 3 Bayesian Intelligence, Monash University LPO, Clayton, Melbourne, Victoria, Australia; Natural Resources Canada, CANADA

## Abstract

Daily flight activity patterns of forest insects are influenced by temporal and meteorological conditions. Temperature and time of day are frequently cited as key drivers of activity; however, complex interactions between multiple contributing factors have also been proposed. Here, we report individual Bayesian network models to assess the probability of flight activity of three exotic insects, *Hylurgus ligniperda*, *Hylastes ater*, and *Arhopalus ferus* in a managed plantation forest context. Models were built from 7,144 individual hours of insect sampling, temperature, wind speed, relative humidity, photon flux density, and temporal data. Discretized meteorological and temporal variables were used to build naïve Bayes tree augmented networks. Calibration results suggested that the *H*. *ater* and *A*. *ferus* Bayesian network models had the best fit for low Type I and overall errors, and *H*. *ligniperda* had the best fit for low Type II errors. Maximum hourly temperature and time since sunrise had the largest influence on *H*. *ligniperda* flight activity predictions, whereas time of day and year had the greatest influence on *H*. *ater* and *A*. *ferus* activity. Type II model errors for the prediction of no flight activity is improved by increasing the model’s predictive threshold. Improvements in model performance can be made by further sampling, increasing the sensitivity of the flight intercept traps, and replicating sampling in other regions. Predicting insect flight informs an assessment of the potential phytosanitary risks of wood exports. Quantifying this risk allows mitigation treatments to be targeted to prevent the spread of invasive species via international trade pathways.

## Introduction

Understanding the abiotic factors that control flight behavior may allow the prediction of flight activity as a function of forecast weather conditions. Such modelling could provide an assessment of the potential for colonization by dispersing bark beetles or wood borers of recently harvested *Pinus radiata* D. Don logs. Understanding the potential for colonization is an important step toward a risk-based approach to managing the phytosanitary requirements for wood exports [1].

New Zealand has 1.7 million ha of managed plantation forests, predominantly (90%) single species, even-age stands of the exotic *P*. *radiata* [2]. Exotic wood boring and bark beetles are sometimes intercepted at New Zealand’s borders, and some have eluded detection, subsequently established, and are now spread widely throughout the country [3]. Three colonizers that are now abundant in plantation forests are two species of bark beetles (Scolytinae), *Hylurgus ligniperda* Fabricius and *Hylastes ater* (Paykull), and one wood borer (Cerambycidae), *Arhopalus ferus* (Mulsant). All three species are saprophytes that colonize recently dead or dying trees [4–6].

Seasonal flight activity has been extensively studied, particularly for *H*. *ligniperda* and *H*. *ater*. Results vary, likely due to the influence of local resources and differences in habitat and abiotic conditions between continents. Reay *et al*. [7] reported spring and autumn flight activity peaks of *H*. *ligniperda* in New Zealand’s central North Island, and Tribe [8] observed an autumnal (April/May) peak in flight activity (April/May) in South Africa, whereas Mausel *et al*. [9] report a single peak of flight activity in spring (September/October) in two regions of Chile and a bi-modal activity pattern in a third region. Comprehensive daily sampling of *H*. *ligniperda* across eight regions in New Zealand indicate the existence of both unimodal and bimodal phenology patterns (Scion, unpublished data). Reay *et al*. [7] report a single autumn peak of *H*. *ater* flight activity in New Zealand, whereas Mausel *et al*. [9] observed both spring and autumn peaks in Chile. Reay *et al*. [7] acknowledged the potential of missing spring flight activity as their sampling began in October. Similarly Sopow *et al*. [10] report a strong autumn peak of activity, but like Reay *et al*. [7] their sampling did not encompass early spring conditions. Recent sampling confirms a strong late summer/autumn emergence of *H*. *ater*, with smaller spring flights in some regions in late August/September (Scion, unpublished data). *Arhopalus ferus* has a single period of flight activity in New Zealand that begins in late October and concludes in late April (Scion, unpublished data).

Despite the attention devoted to the seasonal phenology of these species, little is known regarding the abiotic factors that determine intraday flight activity patterns. Both *H*. *ligniperda* and *H*. *ater* reportedly have a generally crepuscular (active during the hours of dawn and dusk); In New Zealand Kerr *et al*. [11] sampled every three hours over two independent 72-hour periods and found activity peaks for both species from 07:30 to 10:30 and again at 19:30 to 22:30. Similar crepuscular flight patterns have been observed in other Scolydidae, e.g., *Dendroctonus brevicomis* LeConte in California, USA [12], *Pityophthorus juglandis* Blackman in California, USA [13] and *Hylastes nigrinus* (Mannerheim) in Oregon, USA [14]. However, patterns can be seasonal; e.g., in Israel *Orthotomicus erosus* (Wollaston) and *Pityogenes calcaratus* (Eichhoff) were crepuscular in spring and summer but had a unimodal midday peak of flight activity in winter [15].

*Arhopalus ferus* is a nocturnal species; small outdoor cage studies conducted in New Zealand using an infrared movement detector indicated peak activity from 21:00 to 24:00, with some evidence of an additional peak in male activity at dawn in New Zealand [16]. Field trapping in New Zealand at intervals of three hours corroborates these captive studies with maximum trap catch of *A*. *ferus* occurring in 19:30 to 22:30 and 22:30 to 1:30 sampling periods [11]. To date, the abiotic factors that govern the initiation of flight behavior of *H*. *ligniperda*, *H*. *ater*, and *A*. *ferus* have not been quantified. However, morning flight activity of *H*. *ligniperda* and *H*. *ater* was reported by Kerr *et al*. [11] to be coincident with increasing temperature.

Few studies have quantitatively assessed the influence of abiotic factors on the flight of bark beetles and wood borers. Temperature is frequently cited as a strong predictor of flight activity, however, light intensity, wind speed, rainfall, and relative humidity also are reported to influence forest insect flight activity [12, 14, 17, 18]. Although Chen *et al*. [13] observed strong individual relationships between *P*. *juglandis* flight activity and temperature, barometric pressure, light intensity and wind speed, the probability of flight was influenced both collectively and interactively between these four factors [13]; hence, complex interactions make the prediction of flight activity challenging. Such factors may also interact with insects’ chemical host finding cues and subsequently influence trap sensitivity, thus biasing interpretation of flight behavior. For example, low wind speeds could prevent the formation of chemical plumes, precluding effective orientation towards traps by flying insects [18].

Empirical models that quantify the potential risk of infestation by forest insects are needed to develop a risk management framework to guide future application of phytosanitary treatments for export wood products. To support such a framework we developed empirically-based Bayesian network (BN) models to predict the flight activity of three exotic forest insects *H*. *ligniperda*, *H*. *ater*, and *A*. *ferus* in New Zealand as a function of meteorological and temporal factors in recently clear-cut *P*. *radiata* plantations. BNs are graphical networks of variables connected by logical, correlational, or causal relationships quantified by conditional probability tables [19]. They are used widely in ecological and environmental sciences for diagnosis, forecasting, prediction, and other applications [20], including modelling of invasive invertebrates [21]. We chose to use BNs because they express conditions and results as probabilities, particularly for depicting errors of false presence and false absence. This lends BNs to risk analysis and risk management applications [22]. Further, BNs are robust to missing data and to conditions of data multicollinearity and nonlinearity that otherwise violate assumptions in more traditional multivariate modelling approaches. BNs also can be structured from a combination of empirical data and expert knowledge, and can be updated with new information as it comes to hand [23].

## Methods

### Study sites and insect trapping

Four sites were established in *P*. *radiata* plantation forests in Canterbury, New Zealand (S1 Fig, S1 Table). One meteorological station and three flight activity traps were installed at each site. Traps were located equidistant in a circle 40 m from the meteorological station. Each trap consisted of an ethanol and alpha-pinene baited black colored flight intercept panel trap with an electronically controlled stepper motor that rotated a circular carousel to separate trap catch into individual plastic containers (6286PTCL—SQ PET JAR 58MM 233ML, Stowers, New Zealand) on an hourly basis (S2 Fig). The panel trap and plastic containers were coated with an insecticide (cypermethrin), applied at the start of each trapping period, to kill insects and to minimize the potential for any movement between plastic containers by captured individuals. All traps at each site were established two weeks before sampling was initiated to ensure any trap establishment effects, e.g., soil disturbance, did not influence trap efficacy. The trial was run over three non-consecutive periods, with the first in spring (23 September and 14 October 2014) and then twice in summer (19 November to 18 December 2014, and 13 January to 11 February 2015). Each of the focal species has a slightly different phenology, hence sampling periods were spread to encompass periods of known flight activity for each species. Traps were maintained on a daily basis, with insects identified and counted in the field or brought to the laboratory when large numbers were present in a given sample hour. If a trap fault occurred during a 24-hour period, all affected sampling periods were discarded.

### Variables affecting flight activity

A summary of variables used to model flight activity is provided in Table 1. Meteorological data were collected using a 2.5-m metal tower (Scottech, Hamilton, New Zealand). Data from sensors were recorded on a CR1000 (Campbell Scientific, Logan, USA) data logger with measurements taken every minute. Sensors included RM Young wind monitor, model 05103 (RM Young Company, Michigan, USA); Apogee quantum sun calibration sensor, model sq-110 photosynthetic flux density sensor (Apogee Instruments, Logan, USA); CSI temperature and relative humidity probe, model hc2s3 (Campbell Scientific, Logan, USA); and CSI rain gauge, model tb4 (Campbell Scientific, Logan, USA). All meteorological variables were summarized as hourly average, maximum, and minimum values using the R-package xts [24], except rainfall that was calculated as an hourly sum. Time since sunrise and sunset was calculated using the sunriset function in R-maptools [25]. This calculates the time of sunrise (accuracy ± 1minute) corrected for location and atmospheric refraction using the formula of Meeus [26].

**Table 1:** Temporal and abiotic variables used to model the probability of insect flight.

Variable name	Description	Units of measure
*Temporal*		
Day of year	Day of year as integer	Integer
Time since sunrise	Elapsed time between the start of the trapping hour and sunrise. Corrected for geospatial differences in sunrise	Integer (minutes)
Time since sunset	The elapsed time between the start of the trapping hour and sunset. Corrected for geospatial differences in sunset	Integer (minutes)
*Abiotic*		
Maximum hourly temperature	Maximum temperature within the sample hour	°C
Photon flux density	Average radiant flux of solar radiation within the wavelengths of 400 to 700 nm	μmol photons m^−2^s^−1^
Rainfall	Accumulated rainfall within the sample hour	mm
Relative humidity	Average humidity within the sample hour	%
Temperature range	Difference between maximum and minimum temperature within the sampling hour	°C
Wind speed	Average instantaneous wind speed per hour	m^−1^ s^−1^

To define the relationship between meteorological variables and forest insect flight activity, each was regressed across a series of discrete intervals defined using an unsupervised discretization of temperature data, as per the method of Blackburn *et al*. [27]. Variables were discretized into 20 intervals using the unsupervised Minimum Description Length (MDL) method in Weka (vers. 3.6) [28]. *Arhopalus ferus* flight activity was concentrated at low wind speeds, and to improve sensitivity to this we left-shifted the discretization to increase bin numbers at low wind speeds. The maximum observed insect flight activity in each interval was used to fit the relationship with individual meteorological variables. Outliers in models were identified as observations with Cook’s distances >1, using R-nlreg [29]. A Gaussian equation (Y = k×exp(-1/2 * (temperature—μ)^2^/sigma^2^)) was used to fit the relationship between maximum interval flight activity and temperature for all species using the R-nls function. The adjusted R^2^ for the non-linear models was calculated using Wherry’s formula [30]. A negative exponential equation (Y = y0 * exp(wind speed/b) was used to fit the relationship between wind speed and *A*. *ferus* using a generalized non-linear least square, R-nlme [31]. Generalized Additive Models (GAM) with a gamma distribution (inverse link) were used to fit the relationship between flight activity and wind speed, photon flux density (PAR), and relative humidity for *H*. *ligniperda*, using R-mgcv [32]. Graphical model validation tools were used to check the model assumptions of variance homogeneity and normality. The significance of individual GAM models was assessed by a likelihood ratio test of the model against the null, intercept only, model. The adjusted R^2^ was extracted from the model summary. All non-linear modelling was done in R-Version 3.2.2 [33].

### Bayesian network model development and calibration

We developed BN structures for each species using supervised machine learning techniques. The objective of supervised learning is to develop a probabilistic classifier model of a target variable (here, flight activity) based on predictor observations (temperature, time of day, etc.). Classifier models provide a predicted probability distribution over a set of classes (in our case, true or false flight activity), given a set of inputs. The process of developing a BN from data involves three stages: discretization of continuous variables into range states; identification of relationships between variables (that is, the network structure); and parameterization of the probability tables.

For each model training run, the continuous meteorological variables were discretized using a supervised approach relative to the target variable (flight activity) to allocate discrete intervals for subsequent Bayesian network modelling. Using a supervised discretization (the Fayyad *et al*. [34] Minimum Description Length (MDL) method in Weka version 3.6 [28]). The supervised discretization process differs from that used above for the discretization of meteorological variables by providing an information theoretic metric that minimizes the joint entropy between the continuous variable and target variable [34]. It selects a discretization that balances the trade-off between fit to data and model complexity (i.e., more data will justify a greater number of bins). For some variables, the discretization process did not identify useful splits because there was no relationship or the correlation was not strong enough in the data to justify the additional model complexity; in such cases the average value is returned and we excluded such variables from our BN models as they are not informative of flight activity.

We explored three procedures to generate the BN structure: naïve Bayes [35]; CaMML [36], which uses an information theoretic metric to infer causal structure; and Tree Augmented Naïve Bayes (TAN) [37], discussed in detail below. The final network selection was based on a comparison of classification accuracy. In all cases, the TAN structure performed best, and here we report only the results of these models, however models from each of the three approaches are available online at the Australasian Bayesian Network Modelling Society’s BN repository. TAN structure learning involves augmenting the naïve Bayes structure (where all predictive variables are children of the target variable), with dependences between the predictive variables (using a tree structure) to represent correlations between them. TAN and naïve Bayes networks are designed specifically for model prediction and not for denoting causal linkages. We chose this approach because our main objective was to predict insect flight activity. Building TAN structures and conducting calibration and validation assessments of individual models were done using the GeNIe (www.bayesfusion.com/#!genie-modeler/lf73d) Application Programming Interface (API).

Conditional probability tables of TAN models were learnt using the expectation maximization (EM) algorithm [38–40], available in Netica (norsys.com) and GeNIe (and most BN modeling tools). EM iterates to maximize the log likelihood of the BN given the training data, calculating conditional probability values from case data by integrating over missing values [41]. We then conducted sensitivity analysis on the models, using variance reduction [23], to determine the degree to which predictor variables account for flight probability. As more data are collected, it is possible to revise and improve the BN by updating the conditional probabilities or by relearning the BN discretizations and structure. We tested the degree to which each BN model was calibrated to the case files for each species by calculating 5 model performance indices (logarithmic loss and quadratic (Brier) loss, lower values of which denote better model performance; and spherical payoff, Gini coefficient, and area under the ROC curve, higher values of which denote better model performance) and 3 classification error rates (Type I or false positives, Type II or false negatives, and overall confusion error) [42]. Type I errors represented prediction of insect flight when there was no flight (i.e., false positive), Type II errors represented prediction of no flight when flight occurred (i.e., false negative), and overall confusion error is the total error rate over all outcomes. Because Type II error is more egregious in the context of biosecurity policy (wanting to ensure absence of pest insects), we developed a novel ROC curve to depict Type II classification error rates at four probability thresholds of predicting no flight occurrence: 50, 90, 95, and 99%, whereas traditional ROC curves address false positives. We identified the best models on the basis of the lowest type II error rates and the five performance metrics.

### Model validation

One possible concern with calibrating BN structures and parameters to case files by using machine-learning algorithms such as EM is with overfitting the model to the cases observed [43]. Several approaches have been suggested for testing and avoiding overfitting in BNs [44, 45] but none specifically address Type II errors. We devised a novel approach using cross-validation [46] that directly addresses our central concern of minimizing Type II errors as follows. We ran 100 iterations of 4-fold cross-validation analysis, each iteration randomizing the training and testing samples, and relearning the model discretizations, structure, and parameters (see above). We initially ran 3-, 4-, and 5-fold cross validations on each species model and found that error results were nearly identical for each fold, so we settled on 4-fold as the best balance in sample size between training and testing subsets. We recorded the Type I, Type II, and overall confusion error rates for each iteration across a range of probability thresholds from 0 to 1 in 0.001 increments, and then calculated the average, standard deviation, minimum, and maximum error values across all iterations. In addition, we also calculated the number of standard deviations between the calibration and the average of the 100 runs of 4-fold cross validation and plotted the outcome as a means of visualizing any bias between calibration and validation results.

### Model influence runs

We conducted influence runs [47] to determine the maximum influence that each covariate can have on the calculated posterior probability of flight in our model. This was performed by sequentially selecting each state of the input variable, updating the BN, and recording the resulting target variable’s posterior probability. Influence runs quantify the range of effects that each predictor variable set can have on the target outcome, whereas sensitivity analysis tests variance reduction effects from incremental changes of each predictor variable from their normative prior probability value settings.

## Results

### Trap results

**Fig 1:**
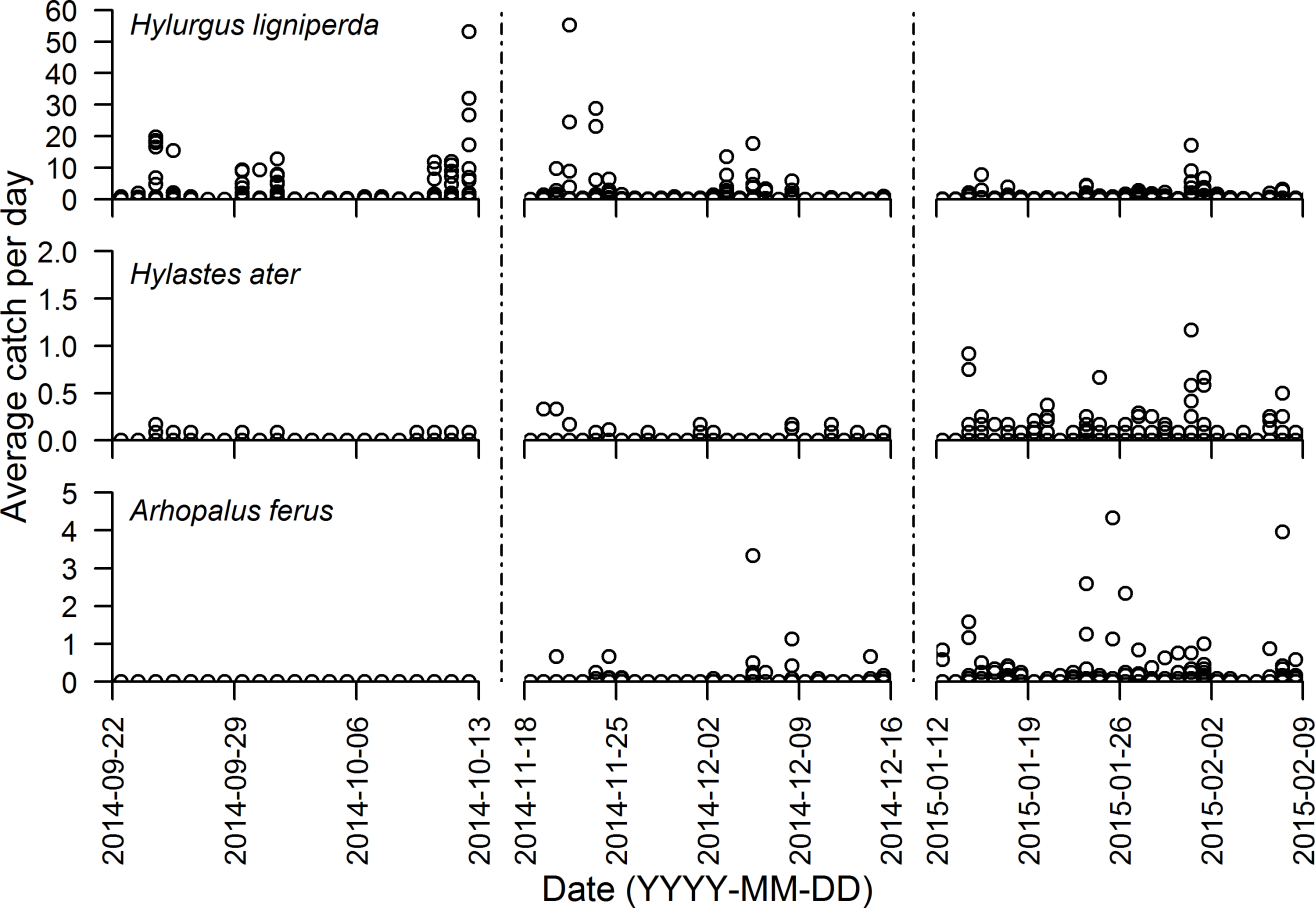
Hourly catch per trap averaged across all traps at the four study sites. Separate panels indicate the three discontinuous time periods when sampling was undertaken.

A total of 10,135 *H*. *ligniperda*, 224 *H*. *ater*, and 553 *A*. *ferus* were trapped during the course of 7,144 individual hours of sampling spread across 78 non-continuous days at the four sites. In total there were 759 (11%), 141 (2%), and 197 (3%) positive trap catch hours of *H*. *ligniperda*, *H*. *ater*, and *A*. *ferus* respectively. Average catch rates per trap varied between the three sample periods. *Hylurgus ligniperda* flight activity was more prevalent in spring and early summer, *H*. *ater* more in late summer, and *A*. *ferus* most pronounced in the second summer sampling period (Fig 1).

**Fig 2:**
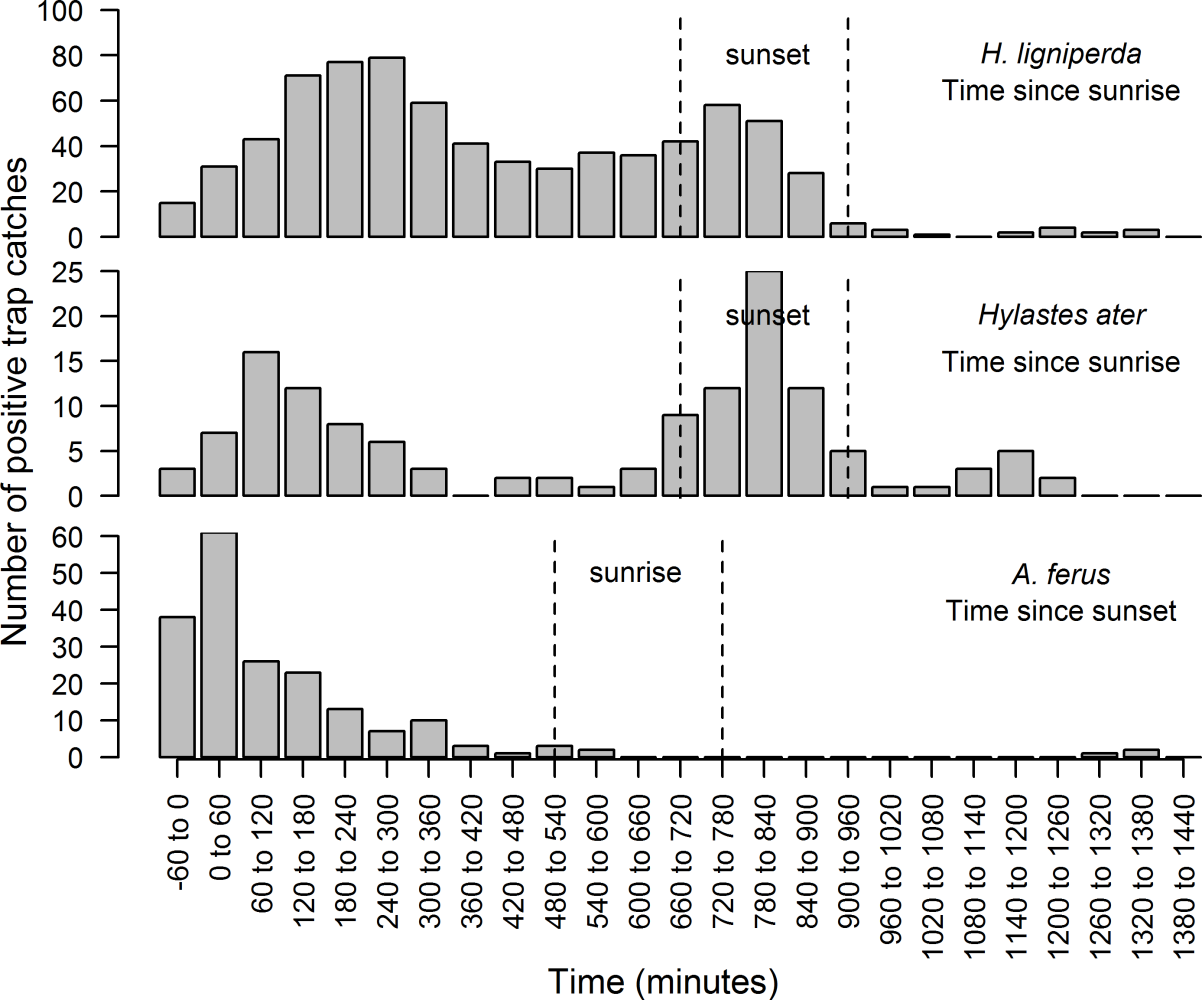
Number of positive trap catch hours as a function of time since sunrise in hourly bins for *H*. *ligniperda* and *H*. *ater* and for time since sunset for *A*. *ferus*. Because day length varied as a function of the day of the year during the study a range is provided that encompasses the period when sunrise or sunset occurred. Dashed lines indicate the period where sunset occurred as a function of time since sunrise for *H*. *ligniperda* and *H*. *ater*. Similarly for the nocturnal *A*. *ferus* these dashed lines indicate the period when sunrise occurred as a function of time since sunset.

Although insecticide was used in trapping containers to reduce the potential for internal movement between containers, 9 individuals were clearly inconsistent with the other 7,144 hourly trap samples and the known behavior of these species. Movement between containers is the most parsimonious explanation. Specific anomalous catches of note were *H*. *ligniperda* activity at -0.7 and 3.6°C that are substantially less than all other cases of positive *H*. *ligniperda* flight activity (S3 Fig) and 7 instances of *A*. *ferus* activity recorded during the day when photon flux density exceeded 500 μmol m^-2^ s^-1^ (S4 Fig). These discrepancies were removed from non-linear regression and BN modeling; however, for completeness they are shown in the overall capture outcomes (Figs 1 and 2, S3–S5 Figs). Trap catch of *H*. *ligniperda* and *H*. *ater* indicated a generally crepuscular flight activity pattern, with *H*. *ater* exhibiting a more pronounced decline in midday flight activity than *H*. *ligniperda* (Fig 2). *Arhopalus ferus* was clearly nocturnal with peak activity occurring within the first hour post sunset (Fig 2).

### Environmental correlates

Average hourly values of each abiotic variable across all sites and time periods are summarized in S6 Fig. Minimum average hourly temperature across all days and sites occurred just before sunrise and peaked at midday. Wind speed was correlated with temperature (r = 0.27, n = 14,138, P<0.001) and peaked in early afternoon. Relative humidity was inversely correlated with temperature (r = -0.66, n = 14,138, p<0.001). Photon flux density, as a proxy for light intensity, was unimodal with respect to time of day with peak intensity at midday. Hourly accumulated rainfall was the most variable abiotic factor between sites (greatest standard error of the mean, S6 Fig) and showed no consistent daily pattern.

A Gaussian relationship fitted the observed flight activity of *H*. *ligniperda* (k = 83.18, μ = 18.57, σ = 3.40), *H*. *ater* (k = 3.16, μ = 18.29, σ = 3.46), and *A*. *ferus* (k = 10.16, μ = 17.28, σ = 3.07) as a function of maximum hourly temperature (S3 Fig). Peak flight activity occurred between 17 and 18°C for all species, with flight activity observed at a minimum hourly maximum temperature of 6.3, 6.3, and 6.4°C for *H*. *ligniperda*, *H*. *ater*, and *A*. *ferus* respectively (excluding outliers discussed above). No flight activity was observed when maximum temperature within an hour exceeded 31.6, 26.3, and 24.7°C for *H*. *ligniperda*, *H*. *ater*, and *A*. *ferus* respectively.

A generalized additive model with a Gamma distribution (Table 2), and a negative exponential model (y0 = 11.93, b = -3.65), best described the relationships between flight activity and wind speed, for *H*. *ligniperda* and *A*. *ferus*, respectively (S5 Fig). Observed flight activity of *H*. *ligniperda* was low at very low wind speeds, but increased quickly with rising wind speed and peaked at 2 m s^-1^. *Arhopalus ferus* was capable of active flight at 15 m s^-1^, however most flight activity of all species occurred at wind speeds < 10 m s^-1^. No clear relationship was observed between the upper bound of *H*. *ater* flight activity and wind speed.

**Table 2:** Results from the final General Additive Models (GAMs) for the flight activity of *H*. *ligniperda*. GAMs have a parametric component and a smoothing part, hence the distinction between parametric coefficients and the smoothing terms. s() = smooth term for a continuous variable, *SE* = standard error of the estimate, *t* = *t*-statistic, *P* = *P*-value, *edf* = estimated degrees of freedom, *F* = *F*-statistic. Wdspd = Wind speed, PAR = Photon flux density, and RH = Relative humidity. Significant values are denoted with P <0.05 = *, P <0.01 = **, P <0.001 = ***.

Parametric coefficients	*Estimate*	*SE*	*t*	*P*	
Intercept					
Wdspd	0.14	0.020	7.01	< 0.001	***
PAR	0.04	7.5 × 10^−3^	5.63	< 0.001	***
RH	0.09	0.015	5.51	< 0.001	***
Approximate significance of smoother terms	*edf*	*F*		*P*	
s(Wdspd)	4.10	8.80		0.016	*
s(PAR)	3.76	3.83		0.022	*
s(RH)	4.05	4.59		0.010	*

A generalized additive model with a Gamma distribution best fitted the relationship between *H*. *ligniperda* and photon flux density and relative humidity (Table 2, S4 Fig). Peak flight activity of *H*. *ligniperda* occurred at very low and intermediate photon flux densities, and at 42% relative humidity. No clear relationship was observed between the upper bound of *H*. *ater* and *A*. *ferus* flight activity and either photon flux density or relative humidity (S4 Fig).

### Bayesian network models

**Fig 3:**
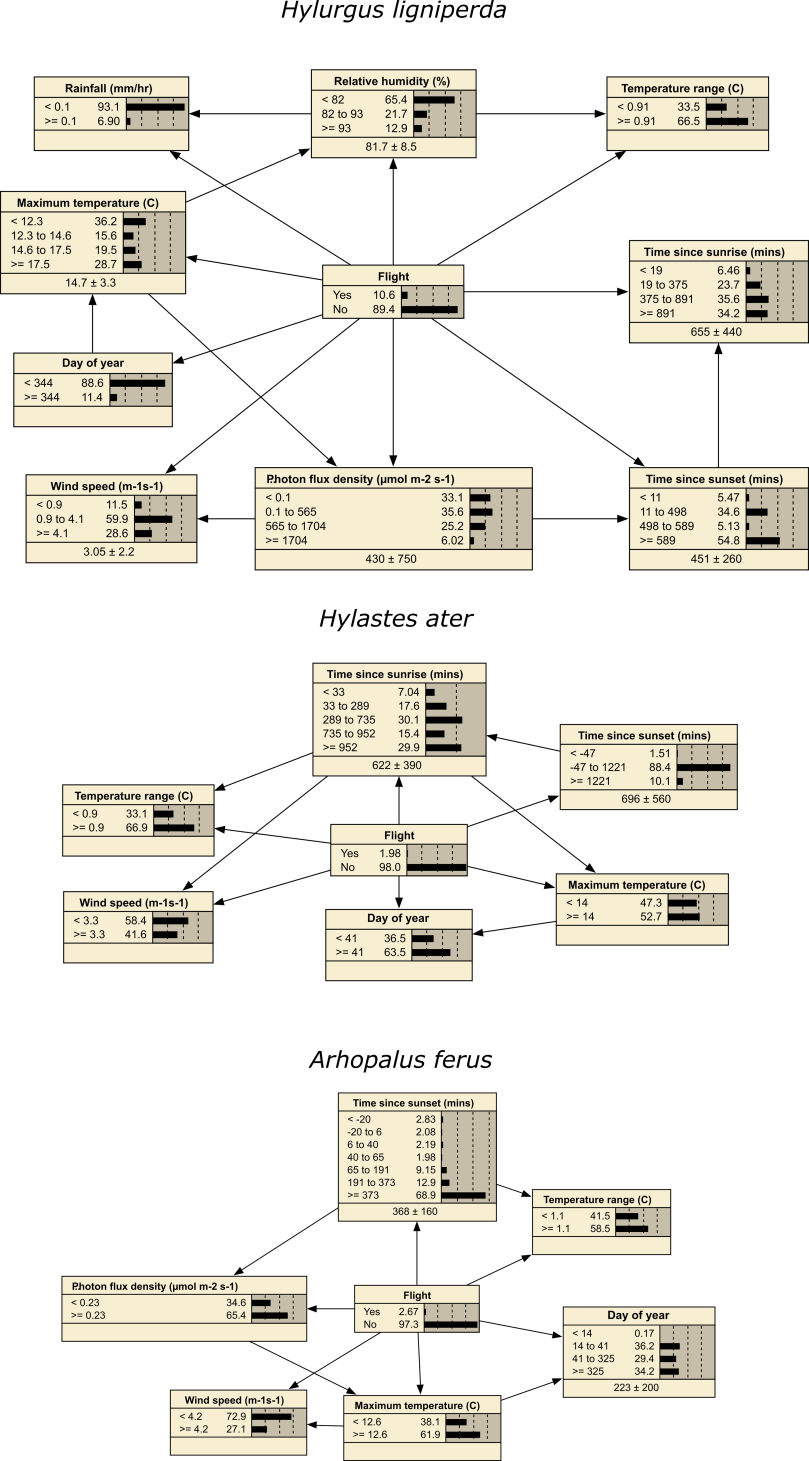
Bayesian networks models of flight activity of three forest insect species.

Bayesian network models are summarized (Table 3) and the best model for each species (Fig 3) was selected by lowest total classification error rate. The probability of *H*. *ligniperda* flight was most sensitive (comprising at least half of all model sensitivity) to maximum hourly temperature and time since sunrise (Table 4). The probability of *H*. *ater* flight was most sensitive to time since sunrise and the time of year (Day of year) (Table 4). The probability of *A*. *ferus* flight was most sensitive to time since sunset (Table 4).

**Table 3:** Description of the best model for each species.

Model	Number of nodes	Number of linkages	Total number of probabilities
*H*. *ligniperda*	10	17	190
*H*. *ater*	7	11	106
*A*. *ferus*	7	11	104

**Table 4:** Sensitivity analysis of the Bayesian network models (Fig 3), showing degree of sensitivity of insect flight probability to each predictor variable.

Variable	Mutual Information	Percent	Variance of Beliefs
*Hylurgus ligniperda*			
Maximum hourly temperature	0.07298	14.9	0.008755
Time since sunrise	0.06282	12.9	0.006895
Time since sunset	0.05696	11.7	0.005719
Photon flux density	0.04507	9.23	0.005316
Relative humidity	0.03162	6.48	0.003277
Temperature range	0.01956	4.01	0.002218
Average wind speed	0.01171	2.4	0.00139
Day of year	0.00813	1.66	0.000802
Rainfall	0.00374	0.766	0.00038
*Hylastes ater*			
Time since sunrise	0.0104	7.42	0.0003
Day of year	0.00939	6.7	0.000262
Time since sunset	0.00466	3.33	0.000205
Maximum hourly temperature	0.00753	5.37	0.000179
Wind speed	0.00331	2.36	8.08E-05
Temperature range	0.00209	1.49	4.97E-05
*Arhopalus ferus*			
Time since sunset	0.05908	33.3	0.004525
Photon flux density	0.03249	18.3	0.001138
Day of year	0.02303	13	0.001088
Maximum hourly temperature	0.00833	4.69	0.000246
Temperature range	0.0029	1.64	0.000106
Wind speed	0.00263	1.48	8.09E-05
Time since sunrise	0.00209	1.49	4.97E-05

### Model calibration

**Fig 4:**
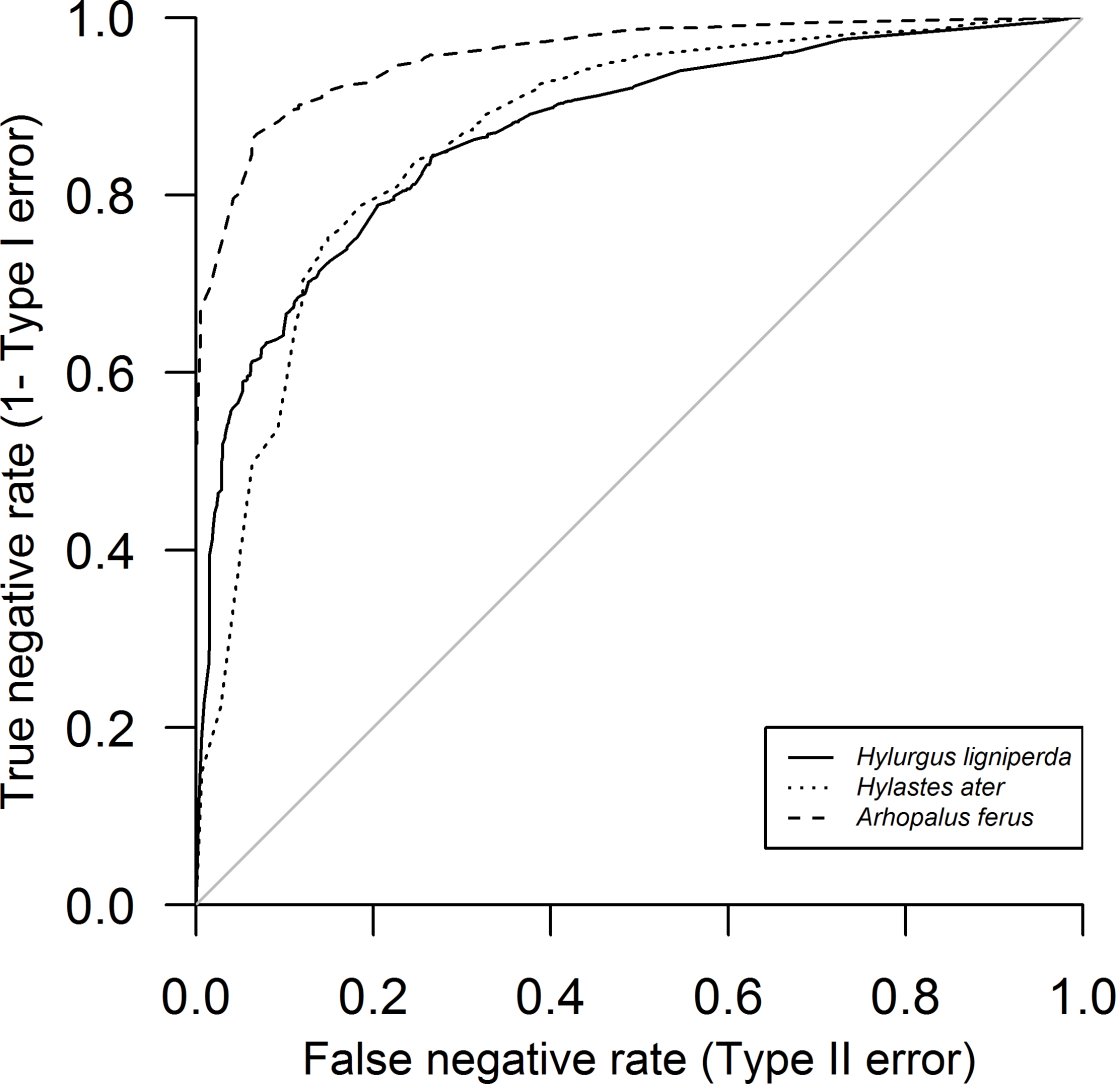
Modified receiver operating curve (ROC) showing model predictions of the true negative state as a function of the type II (false negative) error rate.

BN model calibration results (Table 5) suggest better model fit for *H*. *ater* and *A*. *ferus* than for *H*. *ligniperda*, in particular with the latter showing greater values of logarithmic loss and quadratic loss and slightly lower values of spherical payoff, all denoting poorer model fit. *Arhopalus ferus* had better model fit denoted by higher values of the Gini coefficient and area under the ROC curve (Fig 4). However, consistently across all model thresholds denoting no flight, the *H*. *ligniperda* model had highest Type I and the lowest Type II errors, and the *H*. *ater* model had highest Type II errors. Overall, calibration results suggested that the *H*. *ater* and *A*. *ferus* models had the best fit for low Type I and overall errors, and *H*. *ligniperda* had the best fit for low Type II errors.

**Table 5:** Summary of BN model performance. Model performance is assessed at different predictive thresholds with both calibration (entire dataset) and validation (4-fold cross validation) results presented.

Metric	*Hylurgus ligniperda*				*Hylastes ater*				*Arhopalus ferus*			
Logarithmic loss	0.244				0.074				0.068			
Quadratic loss	0.148				0.035				0.038			
Spherical payoff	0.918				0.982				0.979			
Gini coefficient	0.737				0.738				0.915			
Area under ROC	0.870				0.868				0.958			
*Model threshold predicting no flight*	*50*	*90*	*95*	*99*	*50*	*90*	*95*	*99*	*50*	*90*	*95*	*99*
***Calibration***												
Type I error rate (%)	3	31	39	56	0	5	8	30	1	5	9	15
Type II error rate (%)	73	12	6	2	100	48	38	12	59	23	14	6
Total error rate (%)	10	29	35	50	2	6	8	29	3	6	9	14
***Validation***											
Average type I error rate (%)	2	30	37	55	<1	3	10	37	<1	6	9	17
Average type II error rate (%)	80	17	10	4	100	81	51	22	71	30	20	10
Average total error rate (%)	10	28	35	49	2	5	11	36	3	6	10	17

As expected, across the four model thresholds denoting no flight, Type I errors increased and Type II errors decreased, and overall errors increased. At the most stringent criterion of no flight—that is, when the "no flight" state in the output node achieved ≥ 99% probability—the *H*. *ligniperda* model performed best with minimal Type II errors, but at the cost of a high Type I error rate. The *A*. *ferus* model performed second best with a low Type II error rate but a higher Type I error, and the *H*. *ater* model performed worst with high Type II and Type I errors. No species model achieved <15% simultaneously for Type I and II error rates across the four model thresholds.

### Model validation

**Fig 5:**
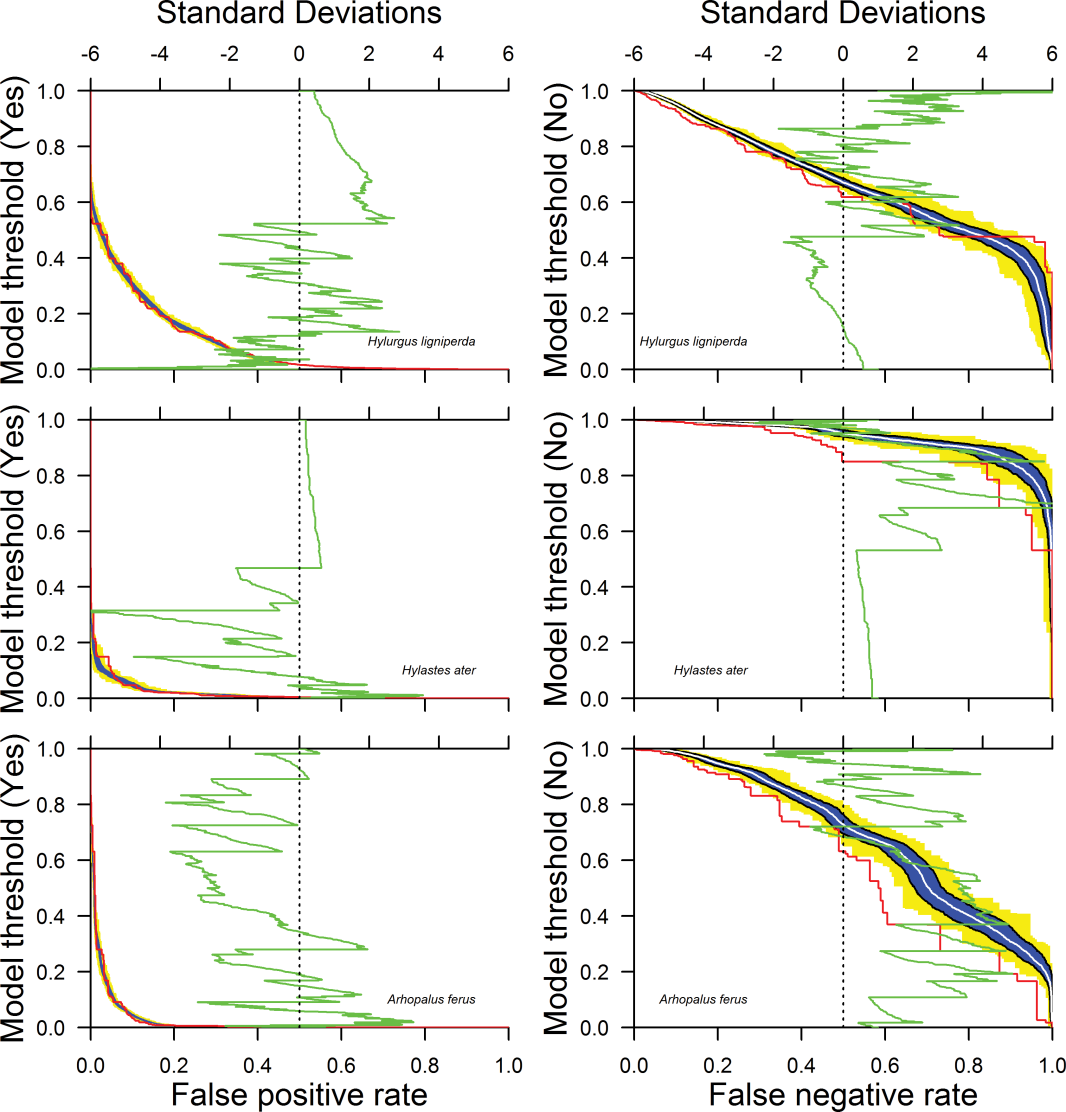
False positive (Type I error) rates as a function of the threshold required for the model to predict ‘Yes’, and false negative (Type II error) rates as a function of the threshold required for the model to predict ‘No’ for *H*. *ligniperda*, *H*. *ater*, and *A*. *ferus*. Red lines indicate the relationship for the calibration dataset (i.e., full casefile), blue shading indicates the range of the first standard deviation for 100 runs of 4-fold cross validation, with the inner white line denoting mean outcomes. Yellow indicates the maximum and minimum values observed during those 100 runs. The green curve represents the number of standard deviations between the calibration and the average of the 100 runs of 4-fold cross validation.

We compared validation to calibration in a novel way. Cross-validation resulted in patterns similar to the calibration results (Table 5, Fig 5) with *H*. *ligniperda* again showing lowest Type II error rates and *H*. *ater* showing highest, and no species model achieving <17% for both Type I and II error rates across the four model thresholds. In general, Type I and II error rates were complementary; reducing one increases the other. This pattern is seen also in comparing calibration and cross-validation results (the green line in Fig 5), where high concordance (low standard deviation difference between the two results) occurred generally with one error type but not the other. False positive rates calculated from the all-data calibration (the red line in Fig 5, left column) generally coincided well with the false positive rates calculated from cross-validation, for all three species. False negative rates from calibration (the red line in Fig 5, right column) coincided well with cross-validation for *H*. *ligniperda* but less so for the other two species, that is, where calibration error rates mostly were less than cross-validation error rates, suggesting some degree of model overfitting.

### Influence runs

**Fig 6:**
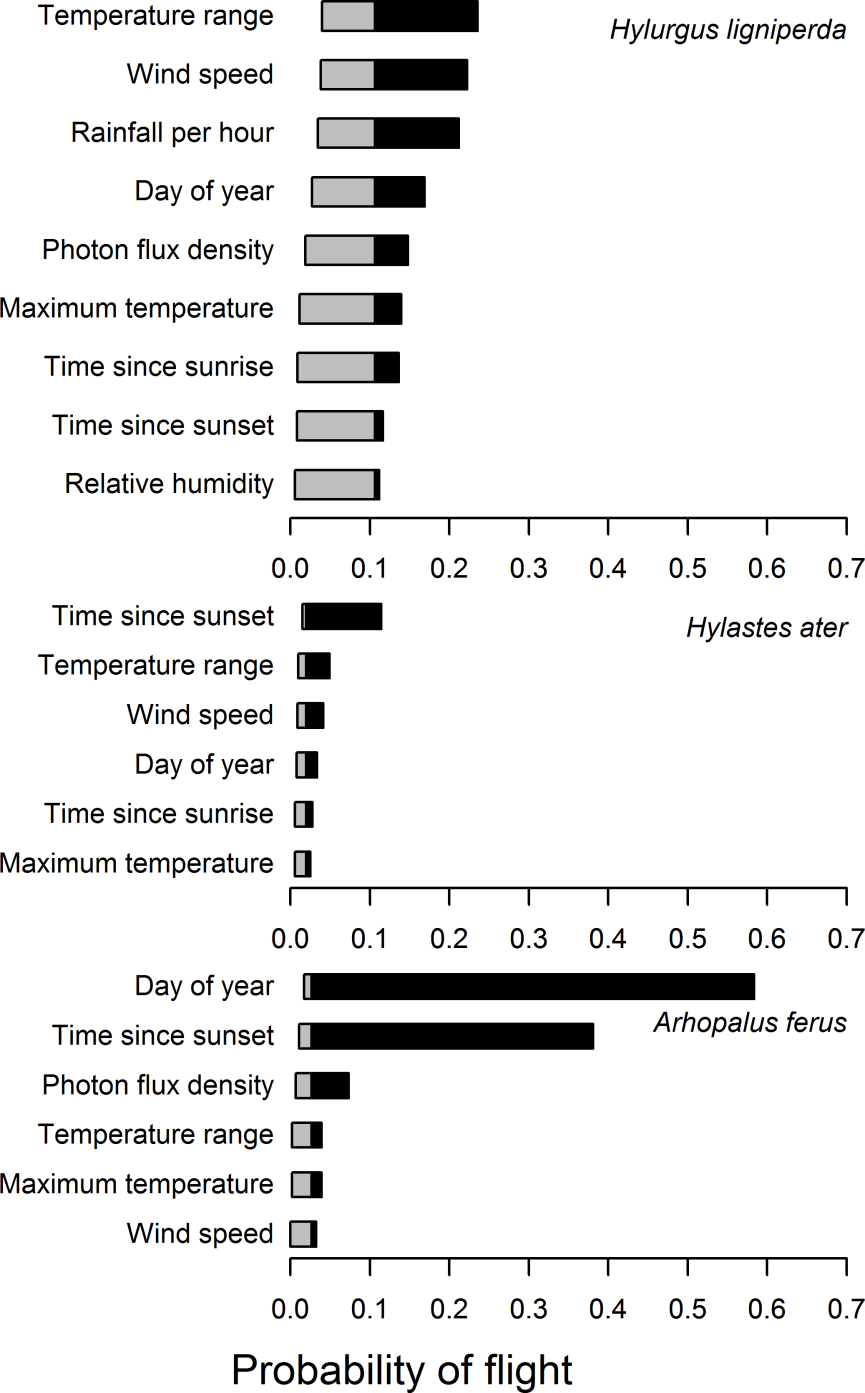
Model influence runs. Presented as tornado diagrams [48] that have a grey and a black portion, to the left and to the right, of the expected ("normative") outcome where all other covariates are set to their prior probability (expected, normative) distributions. Gray portions of the bars show the variables potential range influence on reducing flight probability; black portions show its potential range of influence on increasing flight probability. Variables are sorted in order of decreasing overall influence for each model.

Influence runs (Fig 6) suggest that a number of variables are individually associated with decreased flight probability of all three species. However, only a few variables have major influence on increased flight probability: increasing maximum hourly temperature (≥ 17.5C) and moderate time since sunrise (11–498 min) for *H*. *ligniperda*; shorter time since sunset (sampling hours that began <47 minutes before actual sunset) for *H*. *ater*; and lower days of the year (<14) and shorter time since sunset (sampling hours that began 20 min before to 6 min after actual sunset) for *A*. *ferus*. Other variables also are associated with increased flight probability for all three species, although to lesser degrees.

## Discussion

Chen *et al*. [13] highlight the importance of first and second order interactions among multiple environmental factors as regulators of flight activity in *P*. *juglandis*, the invasive walnut twig borer. Our BN models provide an alternative approach to model the effects of multiple interacting abiotic factors (both meteorological and temporal) on insect flight activity, and thereby determining the overall and individual explanatory power of predictor variables, although results varied amongst species.

In our best performing BN models, *Hylurgus ligniperda* flight activity was most sensitive to maximum hourly temperature, photon flux density, and time since sunrise and sunset (Table 4). Kerr *et al*. [11] also observed temporal patterns with distinct morning and evening peaks of *H*. *ligniperda* and *H*. *ater* activity during short-term (two periods of three-hourly sampling over three days) summer monitoring periods. Similarly, meteorological factors and time of day were key determinants of the *P*. *juglandis* flight activity, as reported by Chen *et al*. [13]. Such concordance highlights the need to consider multivariate models to predict flight activity patterns.

Sensitivity of *H*. *ater* and *A*. *ferus* flight activity to meteorological variables was low compared to the effect of time since sunrise and sunset and the day of the year, although *H*. *ater* flight activity was moderately sensitive to maximum hourly temperature (Table 4). Short-term activity studies of individual caged cerambycids have demonstrated a range of temporal patterns including diurnal activity, e.g., *Callidiellum rufipenne* (Motchulsky) [49] and *Gaurotes virginea* (L.) [50], nocturnal activity, e.g., *A*. *ferus* [16], and cathemeral activity, e.g., *Semanotus bifasciatus* (Motschulsky) [49] and *Nadezhdiella cantori* (Hope) [51]; however more precise prediction of flight as a function of meteorological factors is currently lacking. The overall low sensitivity of flight activity of *H*. *ater* and *A*. *ferus* to meteorological variables in our study may reflect (1) the small fraction of positive flight activity hours within the calibration dataset, and (2) lower attractiveness of *H*. *ater* and *A*. *ferus* to alpha-pinene and ethanol baited panel traps relative to *H*. *ligniperda*. Improving trap sensitivity is one of the most important options to reduce Type II modelling errors of flight activity for these species (see uncertainties below). To test the effect of trap sensitivity on the performance of Bayesian network models requires additional datasets from multiple trap types. Model performance could then be evaluated and potentially enhanced by adding an additional node for trap type.

### Uncertainty as useful information

Our models are built from field data collected from one region (Canterbury) of New Zealand. The robustness of applying the models to other regions likely depends on the similarity of environmental conditions (especially weather patterns and climate regimes) to the Canterbury field site. New Zealand has complex weather patterns with strong gradients in precipitation from west to east. The most similar meteorological regions to Canterbury are Marlborough, Wairarapa, Hawke’s Bay and East Cape [52]; however, the robustness of models between regions would need to be verified by field sampling. The wetter regions down the west coasts of both islands are likely to be most dissimilar to Canterbury [52].

Prediction of flight activity is inherently complex with many interacting factors, e.g., temperature, light, and wind speed, influencing flight [53]. In such circumstances a BN modeling approach is advantageous in that it explicitly shows the degree of uncertainty in the prediction (in this case the flight activity outcome states), denoted as the distribution of posterior probabilities [42]. Because we can assess the implications of uncertainty and error as they propagate through the network, we can identify predictor variables least well understood that could have major influences on prediction outcomes. We identified such variables in our use of sensitivity tests and influence runs, which can be part of a broader assessment of the value of further information [54, 55] given monitoring and study costs and the degree to which they could reduce model error, particularly Type II error. Also, the BN models can be incrementally updated and improved by incorporating new data using the EM algorithm and, where warranted, by restructuring the networks.

### Potential effect of sampling errors on model performance

Parameter uncertainty and prediction accuracy of our flight activity models depend on sampling bias, specifically the sensitivity and specificity of the flight intercept panel traps we used to sample flying beetles. The species we modelled are not known to possess long-range aggregation or sexual pheromone communication systems and our traps relied on kairomones (host volatiles) of ethanol and alpha-pinene as attractants. Host volatiles are generally less powerful than pheromones, and that reduces the sensitivity of traps [56]. Lower trap sensitivity increases the likelihood that our model would predict that conditions are not suitable for flight even though flight occurred, leading to a Type II error. Low trap sensitivity is one potential explanation for the high Type II error rates when the model threshold to predict ‘No’ is low. Increasing the models' predictive threshold partially compensates for this error, but in the absence of data on true detectability, prediction uncertainty remains. Increasing trap sensitivity may reduce type II errors, however more sensitive traps have the potential to sample flying insects from a larger area [57]. Increased sample areas may then be less representative of abiotic prediction variables measured by the meteorological station. The impact of this trade-off between trap sensitivity and model performance is currently unknown.

### Implications for risk management

Prediction of forest insect flight activity over time provides an opportunity to estimate the potential rate of post-harvest log colonization by insects during storage in the forest, at processing sites, or at ports prior to export. Understanding such risks allows phytosanitary treatments to be targeted effectively, thus reducing the potential for on-going spread of such species via international trade. To do this requires an understanding of the form of the relationship between individual log colonization rates and flight activity. Probability of flight must be combined with the infestation rate to make an assessment of overall phytosanitary risk. The form of this relationship is currently unknown, but it is likely to depend on the volume of logs in a given area. Larger concentrations of logs increase the odor plume that will attract beetles to a site, however they also dilute the instantaneous population of flying insects amongst a greater volume of logs, hence reducing infestation rate. Furthermore, knowledge of the ecology of individual species can be used as a management tool to modify the form of the relationship and reduce the likelihood of log colonization. For example, stacking logs above the ground will reduce the rate of colonization irrespective of flight activity, as *H*. *ligniperda* and *H*. *ater* are known to prefer logs in ground contact [9]. On a wider scale, understanding landscape configurations of forest age classes as dispersal corridors or barriers can inform on occurrence of forest insect species [58].

Evidence that conditions were not suitable for insect flight from the time of harvest to export could be used to support the case for temporal periods of low pest pressure when phytosanitary treatments are not necessary, as the risk of log colonization is below the maximum pest limit of importing countries. Setting an appropriate threshold for log exports requires an understanding of the potential risk of entry, establishment, and spread of a specific pest in an importing country [59]. Risk is generally gauged by the probability of an adverse event times its consequence [60]. Our results provide the first part of a fuller pest risk assessment which would entail applying the flight probabilities from our models to further analyses of infestation rates of trees and logs, and then applying those rates to utility estimates of the costs of infestation that pertain to phytosanitary treatments and monitoring as incurred by the wood products export industry. Such scientific assessments might then be used to inform policy and management who would set acceptable risk levels, particularly for false negatives [61]—predicted absence of insect flight activity when in fact insects activity occurred–that have consequences for maintaining export market access.

Selection of acceptable false negative rates has cost implications for export standards and phytosanitary treatments, and also policy implications for releasing potentially infested wood products to trading partners. As an example, in dealing with such decisions, some export sectors use specific sampling protocols to identify acceptable risk levels, e.g., no infested items within a sample of 600 provides an assurance (with 95% confidence) that the infestation rate is less than 0.5% [62]. Such a monitoring framework could be based on a specific risk attitude, market implications of false negatives, an acceptable level of detection, and information on overall infestation rates. In this way, key uncertainties in risk analysis, particularly with false negatives, can play important roles in informing and guiding policy [63].

Our models pertain to environmental correlates of insect flight activity, namely temperature, wind speed, humidity, and other variables that are mediated by forest stand structure, age, proximity to existing stands, and topographic location. Results of the influence analyses (Fig 6) suggest that flight activity could be variously lowered for the three insect species by storing harvested logs in areas with lower maximum temperature, relative humidity, wind speed, and direct solar radiation. Such conditions occur within even-age *Pinus radiata* forest stands >15 years old, with canopy closure >20% that can intercept wind and solar insolation. Hence, temporary in-forest storage of logs is best confined to cut blocks sufficiently small in size that are embedded in mature stands. This conclusion is supported by Mausel *et al*. [9] observation that logs stored in mature stands had lower levels of *H*. *ligniperda* and *H*. *ater* colonization.

Additional steps could refine our models. This includes additional flight activity data to improve model performance and generalize its application to wider geographic regions. An economic "value of information" analysis would assist this by focusing effort on predictor variables that are least well understood but have the most influence on prediction outcomes.

Finally the models should be field tested in an operational framework whereby flight activity is predicted and infestation of recently harvested logs are monitored. Such analysis can be used within a risk management framework to help inform future phytosanitary treatment needs for the global movement of wood commodities, specifically to determine areas of low pest prevalence and to calculate maximum post-fumigation exposure risk of insect occurrence and infestation.

Our approach has relevance, in that it can be applied to fundamental ecological studies of specific organisms and to specific applied ecological problems, including aspects of the invasion process. We recommend to readers that Bayesian networks should be applied more broadly in the management of complex ecological problems. This recommendation is based on their ability to utilize both quantitative and qualitative data simultaneously, their robustness to missing data and conditions of data multicollinearity and nonlinearity that can otherwise violate assumptions in more traditional multivariate modelling, and their ease of adoption by decision makers due to their interactive, graphical interface.

## Supporting information

S1 Fig Location of study sites in Canterbury, New Zealand. Green shading indicates areas of exotic plantation forests.
(PDF)

S2 Fig Separator trap used in study. (PDF)

S3 Fig Average catch per trap per hour as a function of maximum hourly temperature. Red symbols indicate the maximum trap catch per interval, solid blue symbols represent model outliers with a Cook’s distance > 1, and open blue symbols indicate suspect trap catch removed from analysis (see results). The adjusted *R*^2^ provides an estimate of the variance explained by the non-linear curve fitting and are only provided when a non-linear response is identified.
(PDF)

S4 Fig Average catch per trap per hour as a function of photon flux density and relative humidity. Red symbols indicate the maximum trap catch per interval, solid blue symbols represent model outliers with a Cook’s distance < 1, and open blue symbols indicate suspect trap catch removed from analysis (see results). Model predictions are only shown when a significant relationship was present between the predictor and response variable. The adjusted R^2^ provides an estimate of the variance explained by the non-linear curve fitting and are only provided when a non-linear response is identified.
(PDF)

S5 Fig Average catch per trap per hour as a function of wind speed. Red symbols indicate the maximum trap catch per interval, solid blue symbols represent model outliers with a Cook’s distance < 1, and open blue symbols indicate suspect trap catch removed from analysis (see results). Model predictions are only shown when a significant relationship was present between the predictor and response variable. The adjusted R^2^ provides an estimate of the variance explained by the non-linear curve fitting and are only provided when a non-linear response is identified.
(PDF)

S6 Fig Mean hourly meteorological conditions as a function of the time of day averaged across all sites. The grey polygon defines the standard error of the mean for each hourly measurement.
(PDF)

S1 Table Location of individual traps in the four study sites. Two sites were established in Ashley Forest, the first on McGibbons Rd in a recently clear-felled stand with a predominant southeast aspect at 300 m elevation. The second site was on Mt Grey Rd also in a recently clear-felled stand, but with a predominantly eastern aspect at 381m elevation. The third site was on a flat, recently clear-felled site in the West Melton Forest Rd at 104 m elevation. The fourth site was on a flat, recently clear-felled site in McLeans Forest at 59 m elevation. As recent clearfells all sites had no limited to no forest structure, i.e., newly planted sites with 0.3 m seedlings or young trees up to 0.7 m.
(PDF)

S2 Table Title and description of nodes used in Bayesian network models of *Hylurgus ligniperda*, *H*. *ater* and *Arhopalus ferus* flight activity. N/A = the node does not pertain to the species model.
(PDF)

S3 Table Conditional probability tables for each node in the Bayesian network model of *Hylurgus ligniperda* flight activity as discretised from case data using the expectation maximization algorithm. S3A Table. Conditional probability table for node day of year. S3B Table. Conditional probability table for node relative humidity (%).S3C Table. Conditional probability table for node temperature range (°C). S3D Table. Conditional probability table for node rainfall (mm/hr). S3E Table. Conditional probability table for node maximum temperature (°C). S3F Table. Conditional probability table for node time since sunrise (mins). S3G Table. Conditional probability table for node Photon flux density (μmol photons m^−2^s^−1^). S3H Table. Conditional probability table for node Wind speed (m^-1^s^-1^). S3I Table. Conditional probability table for node time since sunset (mins).
(PDF)

S4 Table Conditional probability tables for each node in the Bayesian network model of *Hylastes ater* flight activity as discretised from case data using the expectation maximization algorithm. S4A Table. Conditional probability table for node flight. S4B Table. Conditional probability table for node temperature range (°C). S4C Table. Conditional probability table for node wind speed (m^-1^s^-1^). S4D Table. Conditional probability table for node day of year. S4E Table. Conditional probability table for node maximum temperature (°C). S4F Table. Conditional probability table for node maximum temperature (°C). S4G Table. Conditional probability table for node time since sunrise (mins).
(PDF)

S5 Table Conditional probability tables for each node in the Bayesian network model of *Arhopalus ferus* flight activity as discretised from case data using the expectation maximization algorithm. S5A Table. Conditional probability table for node. S5B Table. Conditional probability table for node time since sunrise (mins). S5C Table. Conditional probability table for node Photon flux density (μmol photons m^−2^s^−1^). S5D Table. Conditional probability table for node wind speed (m^-1^s^-1^). S5E Table. Conditional probability table for node maximum temperature (°C). S5F Table. Conditional probability table for node day of year. S5G Table. Conditional probability table for node temperature range (°C).
(PDF)
