# Supplementary material for: Predicting forest insect flight activity: A Bayesian network approach
Source: PLoS One. 2017 Sep 27;12(9):e0183464. doi: 10.1371/journal.pone.0183464 (PMC5617153; doi:10.1371/journal.pone.0183464)
Supplement: S1 Fig — Green shading indicates areas of exotic plantation forests. (PDF) [file pone.0183464.s001.pdf]

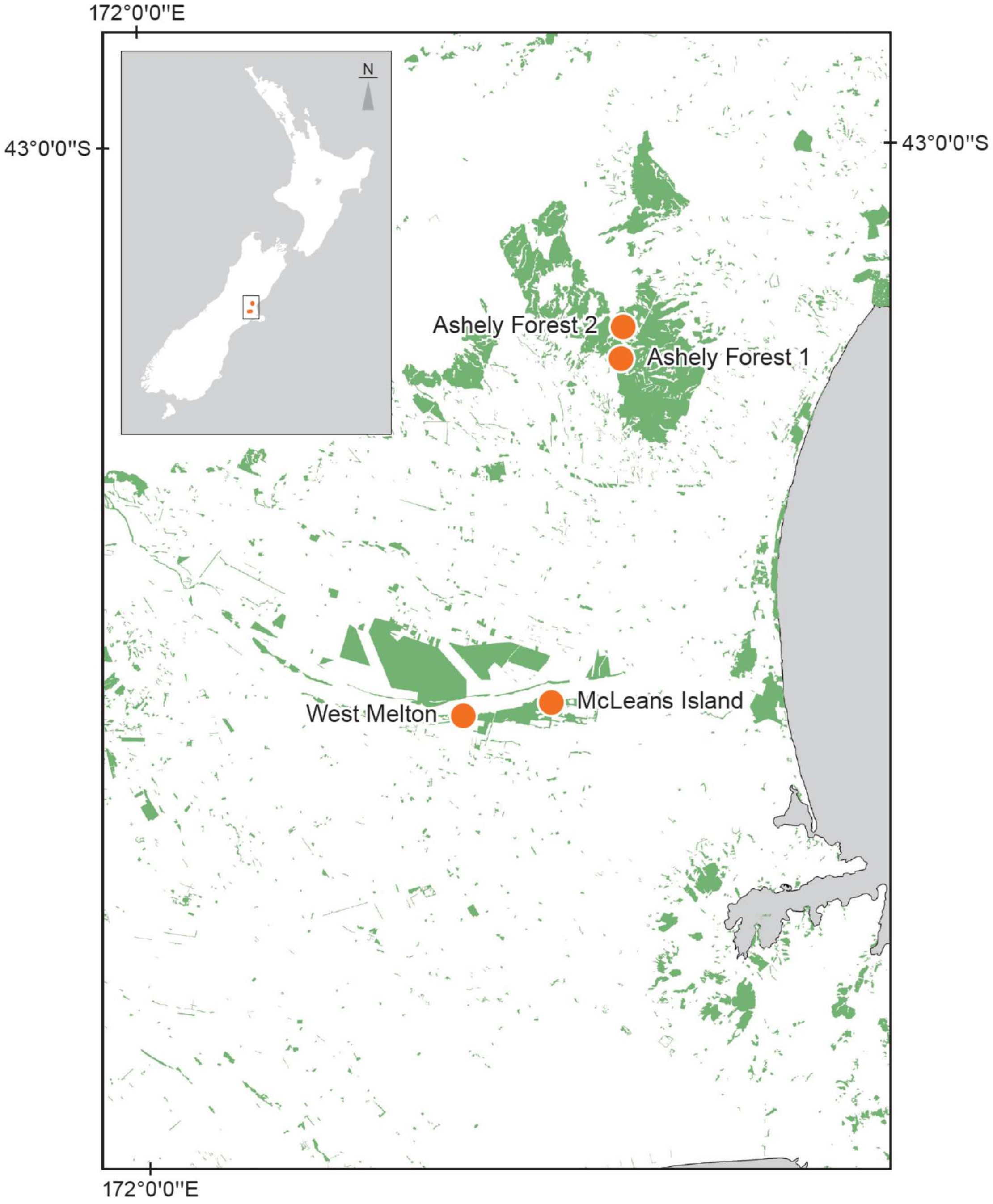

Figure S1. Location of study sites in Canterbury, New Zealand. Green shading indicates areas of exotic plantation forests.
