# Supplementary figures and images for: Predicting forest insect flight activity: A Bayesian network approach
Source: PLoS One. 2017 Sep 27;12(9):e0183464. doi: 10.1371/journal.pone.0183464 (PMC5617153; doi:10.1371/journal.pone.0183464)

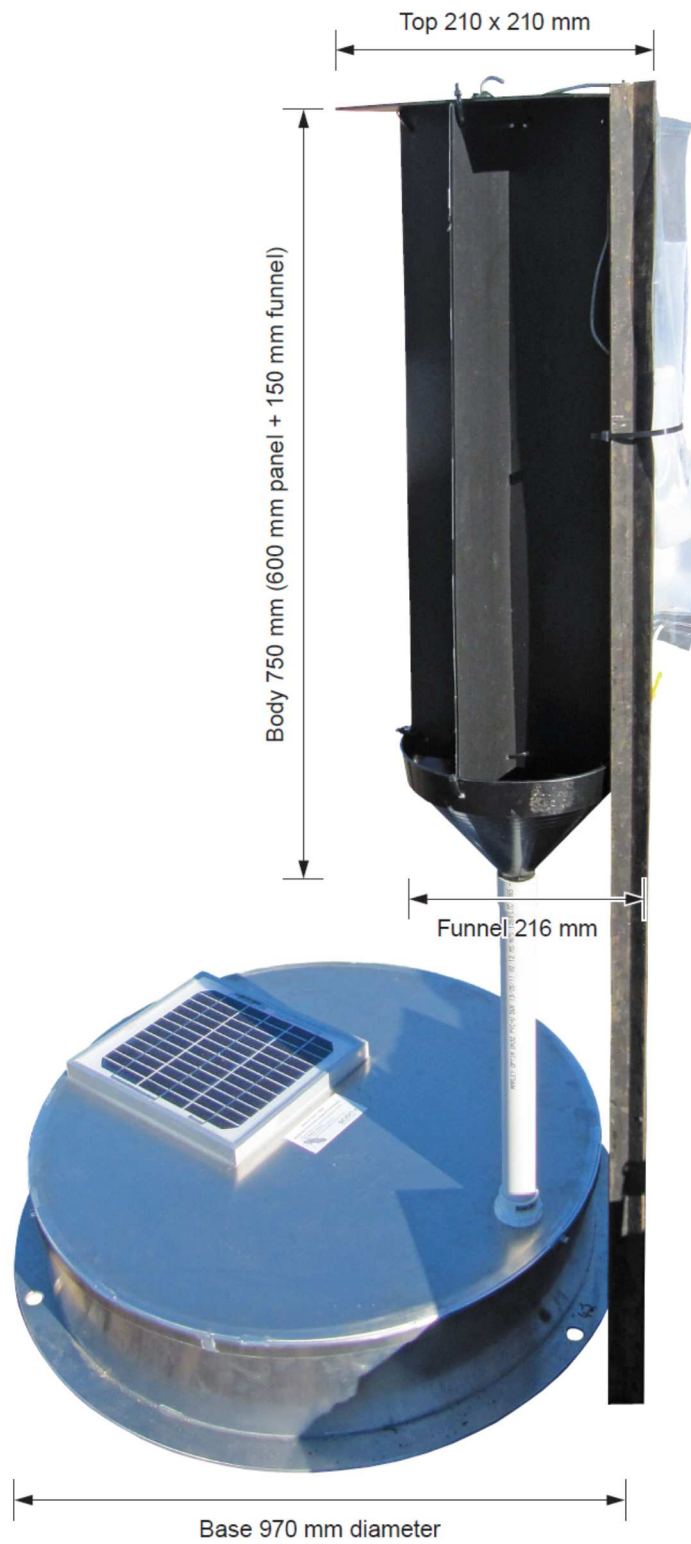

Figure S2. Separator trap used in study

Supplement: S2 Fig — (PDF) [file pone.0183464.s002.pdf]
