# Supplementary material for: Predicting forest insect flight activity: A Bayesian network approach
Source: PLoS One. 2017 Sep 27;12(9):e0183464. doi: 10.1371/journal.pone.0183464 (PMC5617153; doi:10.1371/journal.pone.0183464)
Supplement: S4 Fig — Red symbols indicate the maximum trap catch per interval, solid blue symbols represent model outliers with a Cook’s distance < 1, and open blue symbols indicate suspect trap catch removed from analysis (see results). Model predictions are only shown when a significant relationship was present between the predictor and response variable. The adjusted R2 provides an estimate of the variance explained by the non-linear curve fitting and are only provided when a non-linear response is identified. (PDF) [file pone.0183464.s004.pdf]

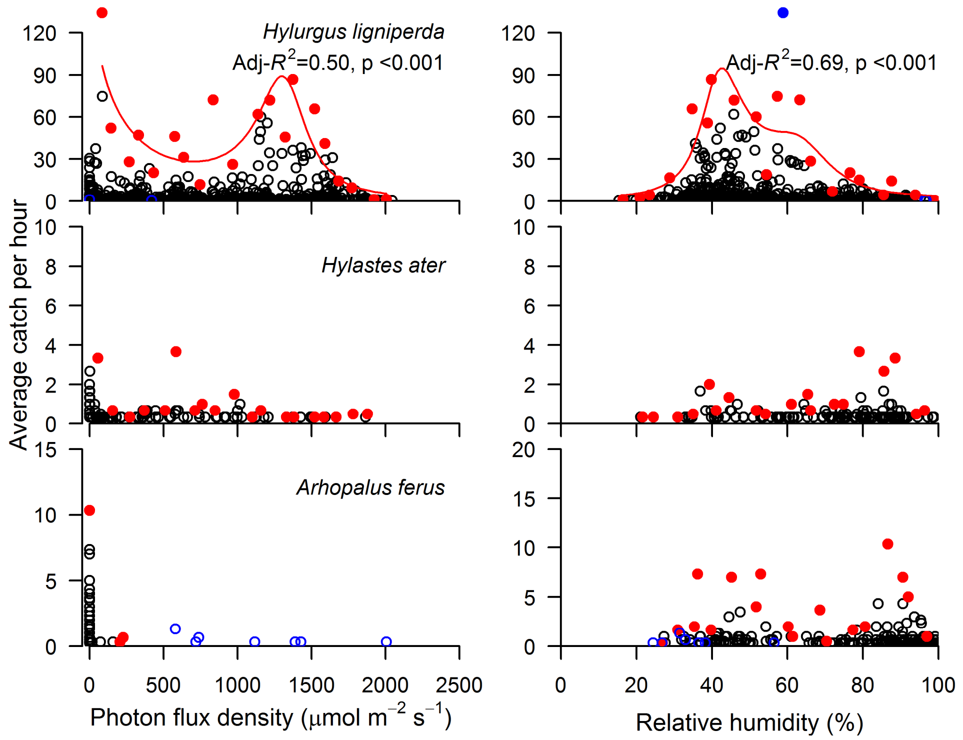

Fig S4. Average catch per trap per hour as a function of photon flux density and relative humidity.

Red symbols indicate the maximum trap catch per interval, solid blue symbols represent model outliers with a Cook's distance < 1, and open blue symbols indicate suspect trap catch removed from analysis (see results). Model predictions are only shown when a significant relationship was present between the predictor and response variable. The adjusted R^2 provides an estimate of the variance explained by the non-linear curve fitting and are only provided when a non-linear response is identified.
