# Supplementary material for: Predicting forest insect flight activity: A Bayesian network approach
Source: PLoS One. 2017 Sep 27;12(9):e0183464. doi: 10.1371/journal.pone.0183464 (PMC5617153; doi:10.1371/journal.pone.0183464)
Supplement: S6 Fig — The grey polygon defines the standard error of the mean for each hourly measurement. (PDF) [file pone.0183464.s006.pdf]

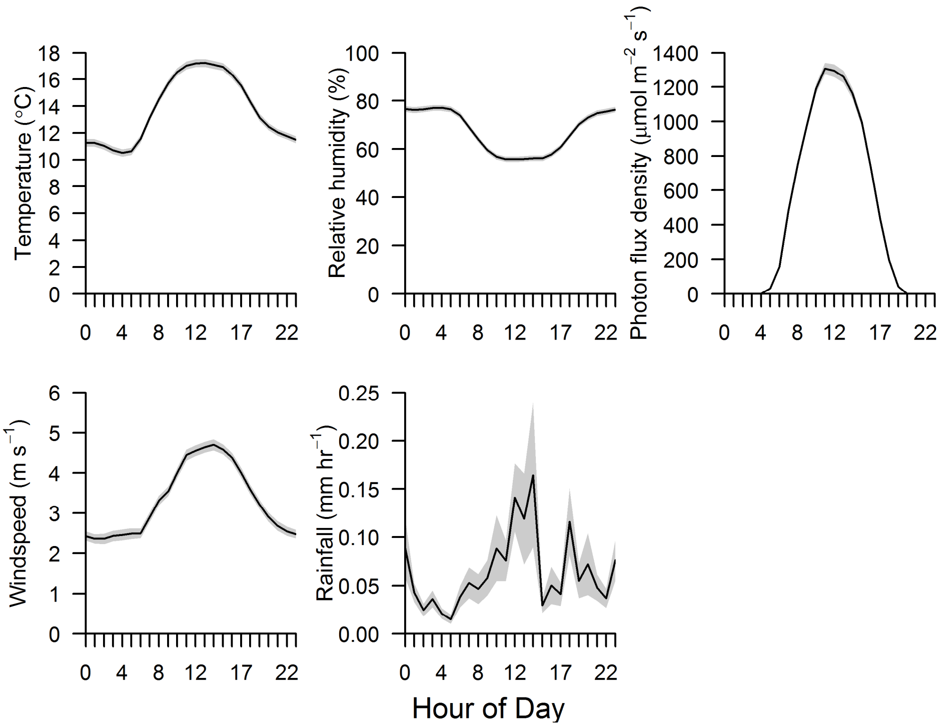

Figure S6. Mean hourly meteorological conditions as a function of the time of day averaged across all sites. The grey polygon defines the standard error of the mean for each hourly measurement.
