# Supplementary material for: Predicting forest insect flight activity: A Bayesian network approach
Source: PLoS One. 2017 Sep 27;12(9):e0183464. doi: 10.1371/journal.pone.0183464 (PMC5617153; doi:10.1371/journal.pone.0183464)
Supplement: S1 Table — Two sites were established in Ashley Forest, the first on McGibbons Rd in a recently clear-felled stand with a predominant southeast aspect at 300 m elevation. The second site was on Mt Grey Rd also in a recently clear-felled stand, but with a predominantly eastern aspect at 381m elevation. The third site was on a flat, recently clear-felled site in the West Melton Forest Rd at 104 m elevation. The fourth site was on a flat, recently clear-felled site in McLeans Forest at 59 m elevation. As recent clearfells all sites had no limited to no forest structure, i.e., newly planted sites with 0.3 m seedlings or young trees up to 0.7 m. (PDF) [file pone.0183464.s007.pdf]

Table S1: Location of individual traps in the four study sites

Two sites were established in Ashley Forest, the first on McGibbons Rd in a recently clear-felled stand with a predominant southeast aspect at 300 m elevation. The second site was on Mt Grey Rd also in a recently clear-felled stand, but with a predominantly eastern aspect at 381m elevation. The third site was on a flat, recently clear-felled site in the West Melton Forest Rd at 104 m elevation. The fourth site was on a flat, recently clear-felled site in McLeans Forest at 59 m elevation. As recent clearfells all sites had no limited to no forest structure, i.e., newly planted sites with 0.3 m seedlings or young trees up to 0.7 m.

Forest	Location	Forest Compartment	Harvest Year	NZTM East	NZTM North
Ashley Forest	McGibbons Rd	29	2012	1562257	5219568
Ashley Forest	McGibbons Rd	29	2012	1562274	5219605
Ashley Forest	McGibbons Rd	29	2012	1562216	5219563
Ashley Forest	McGibbons Rd	29	2012	1562232	5219536
Ashley Forest	Mt Grey Rd	62	2012	1562429	5222459
Ashley Forest	Mt Grey Rd	62	2012	1562427	5222418
Ashley Forest	Mt Grey Rd	62	2012	1562415	5222496
Ashley Forest	Mt Grey Rd	62	2012	1562390	5222467
West Melton	Thompsons Rd	12	2012	1547946	5187228
West Melton	Thompsons Rd	12	2012	1547905	5187228
West Melton	Thompsons Rd	12	2012	1547948	5187188
West Melton	Thompsons Rd	12	2012	1547985	5187227
McLean's Island	Norton Ave	28	2011	1555927	5188432
McLean's Island	Norton Ave	28	2011	1555887	5188427
McLean's Island	Norton Ave	28	2011	1555930	5188393
McLean's Island	Norton Ave	28	2011	1555966	5188427
