# Supplementary material for: Predicting forest insect flight activity: A Bayesian network approach
Source: PLoS One. 2017 Sep 27;12(9):e0183464. doi: 10.1371/journal.pone.0183464 (PMC5617153; doi:10.1371/journal.pone.0183464)
Supplement: S2 Table — N/A = the node does not pertain to the species model. (PDF) [file pone.0183464.s008.pdf]

Table S2. Title and description of nodes used in Bayesian network models of *Hylurgus ligniperda*, *H. ater* and *Arhopalus ferus* flight activity. N/A = the node does not pertain to the species model.

**Node title**	**Node description**	**States**		
		Hylurgus ligniperda	*Hylastes ater*	*Arhopalus ferus*
Flight	The occurrence of flight during that hour.	Yes<br>No	Yes<br>No	Yes<br>No
Day of year	The day of the year was expressed as a decimal (001-366).<br>Because the sampling occurred across three discontinuous periods we incorporate the day of the year as a measure of the time of year to indicate if there are seasonal differences to the flight activity.	<344<br>>=344	<41<br>>=41	<14<br>>14 to 41<br>>41 to 325<br>>=325
Maximum temperature (C)	The maximum temperature recorded during the hour. Measurements during the hour were taken every minute.	<12.3<br>>12.3 to 14.6<br>>14.6 to 17.5<br>>=17.5	<14.0<br>>14.0	<12.6<br>>=12.6
Photon flux density (µmol photons $m^{-2}s^{-1}$)	Measure of the average photons in the 400-700nm range per square metre per second during the sampling hour. The average is of observations taken every minute.	<0.1<br>>0.1 to 565<br>>565 to 1704<br>>1704	N/A	<0.23<br>>=0.23
Rainfall (mm/hr)	Cumulative rainfall across the sampling hour.	<0.1<br>>=0.1	N/A	N/A
Relative humidity (%)	The average relative humidity calculated from minute observations during the hour	<82<br>>82 to 93<br>>=93	N/A	N/A
Temperature range (C)	The range (in °C) between the maximum and minimum temperatures recorded during the sampling hour. Measurements during the hour were taken every minute.	<0.91<br>>=0.91	<0.90<br>>=0.90	<1.1<br>>=1.1
Time since sunrise (mins)	The time in minutes since sunrise as measured from the start of the sampling hour. For example, if sunrise was at 5:50am, the trap hour beginning at 6am would have a value of -10 minutes, however the 5am trap hour would have a value of 50 minutes.	<19<br>>19 to 375<br>>375 to 891<br>>=891	<33<br>>33 to 289<br>>289 to 735<br>>735 to 952<br>>=952	N/A
Time since sunset (mins)	The time in minutes since sunset as measured from the start of the sampling hour. For example, if sunset was at 8:30pm, the trap hour beginning at 8pm would have a value of -30 minutes, however the 9pm trap hour would have a value of 30 minutes.	<11<br>>11 to 498<br>>498 to 589<br>>=589	<-47<br>>-47 to 1221<br>>=1221	<-20<br>>-20 to 6<br>>6 to 40<br>>40 to 65<br>>65 to 191<br>>191 to 373<br>>373
Wind speed (m-1s-1)	Average wind speed during the hour, calculated from minute observations.	<0.9<br>>0.9 to 4.1<br>>=4.1	<3.3<br>>=3.3	<4.2<br>>=4.2
