# Supplementary material for: Predicting forest insect flight activity: A Bayesian network approach
Source: PLoS One. 2017 Sep 27;12(9):e0183464. doi: 10.1371/journal.pone.0183464 (PMC5617153; doi:10.1371/journal.pone.0183464)
Supplement: S3 Table — S3A Table. Conditional probability table for node day of year. S3B Table. Conditional probability table for node relative humidity (%).S3C Table. Conditional probability table for node temperature range (°C). S3D Table. Conditional probability table for node rainfall (mm/hr). S3E Table. Conditional probability table for node maximum temperature (°C). S3F Table. Conditional probability table for node time since sunrise (mins). S3G Table. Conditional probability table for node Photon flux density (μmol photons m−2s−1). S3H Table. Conditional probability table for node Wind speed (m-1s-1). S3I Table. Conditional probability table for node time since sunset (mins). (PDF) [file pone.0183464.s009.pdf]

Table S3. Conditional probability tables for each node in the Bayesian network model of *Hylurgus ligniperda* flight activity as discretized from case data using the expectation maximization algorithm

Table S3A. Conditional probability table for node Day of year

Flight	Outcome	
	**< 344**	**>= 344**
Yes	0.970938	0.029062
No	0.876216	0.123784

Table S3B. Conditional probability table for node relative humidity (%)

Flight	Maximum Temperature	Outcome		
		**< 82**	**>82 to 93**	**>= 93**
Yes	< 12.3	0.466667	0.333333	0.200000
Yes	>12.3 to 14.6	0.792453	0.188679	0.018868
Yes	>14.6 to 17.5	0.848958	0.140625	0.010417
Yes	>= 17.5	0.970954	0.029046	2.07E-08
No	< 12.3	0.374411	0.378336	0.247253
No	>12.3 to 14.6	0.679849	0.169491	0.150659
No	>14.6 to 17.5	0.649958	0.252297	0.097744
No	>= 17.5	0.970644	0.026165	0.003191

Table S3C. Conditional probability table for node temperature range (°C)

Flight	Relative humidity	Outcome	
		**< 0.91**	**>= 0.91**
Yes	< 82	0.091703	0.908297
Yes	>82 to 93	0.393443	0.606557
Yes	>= 93	0.888888	0.111112
No	< 82	0.192201	0.807799
No	>82 to 93	0.587761	0.412239
No	>= 93	0.718202	0.281798

Table S3D. Conditional probability table for node rainfall (mm/hr)

Flight	Relative humidity	Outcome	
		< 0.1	>= 0.1
Yes	< 82	0.983988	0.016012
Yes	>82 to 93	0.934426	0.065574
Yes	>= 93	0.777777	0.222223
No	< 82	0.978365	0.021635
No	>82 to 93	0.886348	0.113652
No	>= 93	0.758772	0.241228

Table S3E. Conditional probability table for node maximum temperature (°C)

Flight	Day of year	Outcome			
		< 12.3	>12.3 to 14.6	>14.6 to 17.5	>= 17.5
Yes	< 344	0.039456	0.066667	0.244898	0.648979
Yes	>= 344	0.045455	0.181818	0.545454	0.227273
No	< 344	0.381021	0.157028	0.198568	0.263384
No	>= 344	0.532319	0.234474	0.111534	0.121673

Table S3F. Conditional probability table for node time since sunrise (mins)

Flight	Time since sunset (mins)	Outcome			
		< 19	>19 to 375	>375 to 891	>= 891
Yes	< 11	1.75E-07	1.75E-07	0.947368	0.0526317
Yes	>11 to 498	4.76E-07	4.76E-07	0.142857	0.857142
Yes	>498 to 589	0.944443	5.56E-07	5.56E-07	0.055556
Yes	>= 589	0.003026	0.564296	0.432678	1.51E-08
No	< 11	3.00E-08	3.00E-08	0.729730	0.27027
No	>11 to 498	0.040098	4.09E-09	0.078969	0.880933
No	>498 to 589	0.600575	0.014368	2.87E-08	0.385057
No	>= 589	0.041551	0.404432	0.542013	0.0120037

Table S3G. Conditional probability table for node Photon flux density (µmol photons $m^{-2}s^{-1}$)

Flight	Maximum Temperature (°C)	Outcome			
		< 0.1	>0.1 to 565	>565 to 1704	>= 1704
Yes	< 12.3	0.366666	0.433333	0.200000	3.33E-07
Yes	>12.3 to 14.6	0.094340	0.584905	0.320755	1.89E-07
Yes	>14.6 to 17.5	0.062500	0.421875	0.484375	0.031250
Yes	>= 17.5	0.035270	0.331950	0.547718	0.0850623
No	< 12.3	0.490188	0.434851	0.074961	3.92E-09
No	>12.3 to 14.6	0.390772	0.369115	0.230697	0.0094162
No	>14.6 to 17.5	0.342523	0.333333	0.279866	0.0442774
No	>= 17.5	0.155073	0.228462	0.412891	0.203574

Table S3H. Conditional probability table for node Wind speed ($m^{-1}s^{-1}$)

Flight	Wind speed ($m^{-1}s^{-1}$)	Outcome		
		< 0.9	>0.9 to 4.1	>= 4.1
Yes	< 0.1	0.244444	0.600000	0.155556
Yes	>0.1 to 565	0.066667	0.789474	0.143860
Yes	>565 to 1704	0.005263	0.794737	0.200000
Yes	>= 1704	2.13E-07	0.553191	0.446808
No	< 0.1	0.227018	0.598187	0.174795
No	>0.1 to 565	0.108551	0.651307	0.240142
No	>565 to 1704	0.010578	0.504937	0.484485
No	>= 1704	2.62E-08	0.319372	0.680628

Table S3I. Conditional probability table for node time since sunset (mins)

Flight	Photon flux density (µmol photons $m^{-2}s^{-1}$)	Outcome			
		< 11	>11 to 498	>498 to 589	>= 589
Yes	< 0.1	0.622222	0.377778	2.22E-07	2.22E-07
Yes	>0.1 to 565	0.101754	0.0140351	0.0631579	0.821053
Yes	>565 to 1704	2.63E-08	2.63E-08	2.63E-08	1
Yes	>= 1704	2.13E-07	2.13E-07	2.13E-07	0.999999
No	< 0.1	0.0984031	0.866638	0.0276219	0.00733708
No	>0.1 to 565	0.0465219	0.193177	0.125831	0.634471
No	>565 to 1704	7.05E-09	7.05E-09	7.05E-09	1
No	>= 1704	2.62E-08	2.62E-08	2.62E-08	1
