# Supplementary material for: Predicting forest insect flight activity: A Bayesian network approach
Source: PLoS One. 2017 Sep 27;12(9):e0183464. doi: 10.1371/journal.pone.0183464 (PMC5617153; doi:10.1371/journal.pone.0183464)
Supplement: S4 Table — S4A Table. Conditional probability table for node flight. S4B Table. Conditional probability table for node temperature range (°C). S4C Table. Conditional probability table for node wind speed (m-1s-1). S4D Table. Conditional probability table for node day of year. S4E Table. Conditional probability table for node maximum temperature (°C). S4F Table. Conditional probability table for node maximum temperature (°C). S4G Table. Conditional probability table for node time since sunrise (mins). (PDF) [file pone.0183464.s010.pdf]

Table S4. Conditional probability tables for each node in the Bayesian network model of *Hylastes ater* flight activity as discretized from case data using the expectation maximization algorithm.

Table S4A. Conditional probability table for node flight

**Flight**	
Yes	0.019767
No	0.980233

Table S4B. Conditional probability table for node temperature range (°C)

**Flight**	**Time since sunrise (mins)**	**Outcome**	
		**< 0.9**	**>= 0.9**
Yes	< 33	0.666666	0.333334
Yes	33 to 289	0.057693	0.942307
Yes	289 to 735	0.190477	0.809524
Yes	735 to 952	0.192308	0.807692
Yes	>= 952	0.307693	0.692307
No	< 33	0.262525	0.737475
No	33 to 289	0.133112	0.866889
No	289 to 735	0.183827	0.816173
No	735 to 952	0.530144	0.469857
No	>= 952	0.520057	0.479943

Table S4C. Conditional probability table for node wind speed ($m^{-1}s^{-1}$)

**Flight**	**Time since sunrise (mins)**	**Outcome**	
		**< 3.3**	**>= 3.3**
Yes	< 33	0.666666	0.333334
Yes	33 to 289	0.884615	0.115385
Yes	289 to 735	0.619048	0.380953
Yes	735 to 952	0.769231	0.230769
Yes	>= 952	0.999999	7.69E-07
No	< 33	0.733467	0.266533
No	33 to 289	0.535774	0.464226
No	289 to 735	0.349788	0.650212
No	735 to 952	0.668899	0.331100
No	>= 952	0.756017	0.243983

Table S4D. Conditional probability table for node Day of year

Flight	Maximum Temperature (°C)	Outcome	
		< 41	>= 41
Yes	< 14	0.473684	0.526316
Yes	>= 14	0.803279	0.196721
No	< 14	0.166418	0.833582
No	>= 14	0.532014	0.467986

Table S4E. Conditional probability table for node maximum temperature (°C)

Flight	Time since sunrise (mins)	Outcome	
		< 14	>= 14
Yes	< 33	3.33E-06	0.999997
Yes	33 to 289	0.019231	0.980769
Yes	289 to 735	0.238096	0.761904
Yes	735 to 952	0.134615	0.865385
Yes	>= 952	0.461538	0.538462
No	< 33	0.625251	0.374750
No	33 to 289	0.318636	0.681364
No	289 to 735	0.283028	0.716972
No	735 to 952	0.588517	0.411483
No	>= 952	0.680038	0.319962

Table S4F. Conditional probability table for node maximum temperature (°C)

Flight	Time since sunset (mins)	Outcome		
		< -47	-47 to 1221	>= 1221
Yes		0.085106	0.687943	0.22695
No		0.01373	0.887729	0.098541

Table S4G. Conditional probability table for node time since sunrise (mins)

Flight	Time since sunset (mins)	Outcome				
		< 33	33 to 289	289 to 735	735 to 952	>= 952
Yes	< -47	8.33E-07	8.33E-07	8.33E-07	0.999997	8.33E-07
Yes	-47 to 1221	0.030928	0.536082	0.134021	0.164948	0.134021
Yes	>= 1221	3.12E-07	3.12E-07	0.250000	0.749999	3.12E-07
No	< -47	1.04E-07	1.04E-07	0.072917	0.927083	1.04E-07
No	-47 to 1221	0.080393	0.193652	0.286773	0.097793	0.341389
No	>= 1221	1.45E-08	1.45E-08	0.493469	0.506531	1.45E-08
