# Supplementary material for: Predicting forest insect flight activity: A Bayesian network approach
Source: PLoS One. 2017 Sep 27;12(9):e0183464. doi: 10.1371/journal.pone.0183464 (PMC5617153; doi:10.1371/journal.pone.0183464)
Supplement: S5 Table — S5A Table. Conditional probability table for node. S5B Table. Conditional probability table for node time since sunrise (mins). S5C Table. Conditional probability table for node Photon flux density (μmol photons m−2s−1). S5D Table. Conditional probability table for node wind speed (m-1s-1). S5E Table. Conditional probability table for node maximum temperature (°C). S5F Table. Conditional probability table for node day of year. S5G Table. Conditional probability table for node temperature range (°C). (PDF) [file pone.0183464.s011.pdf]

Table S5. Conditional probability tables for each node in the Bayesian network model of *Arhopalus ferus* flight activity as discretized from case data using the expectation maximization algorithm.

Table S5A. Conditional probability table for node

Flight	
Yes	0.0266629
No	0.973337

Table S5B. Conditional probability table for node time since sunrise (mins)

Flight	Time since sunset (mins)	Outcome						
		< -20	-20 to 6	6 to 40	40 to 65	65 to 191	191 to 373	>= 373
Yes		0.01579	0.294737	0.052632	0.210526	0.247368	0.131579	0.047368
No		0.028691	0.013264	0.02105	0.014562	0.087226	0.128604	0.706603

Table S5C. Conditional probability table for node Photon flux density (µmol photons $m^{-2}s^{-1}$)

Flight	Time since sunset (mins)	Outcome	
		< 0.23	>= 0.23
Yes	< -20	0.999997	3.33E-06
Yes	-20 to 6	~1	1.79E-07
Yes	6 to 40	0.899999	0.100001
Yes	40 to 65	~1	2.50E-07
Yes	65 to 191	~1	2.13E-07
Yes	191 to 373	~1	4.00E-07
Yes	>= 373	1.11E-06	0.999999
No	< -20	0.492462	0.507538
No	-20 to 6	0.978261	0.021739
No	6 to 40	0.979452	0.020548
No	40 to 65	0.990099	0.009901
No	65 to 191	0.971901	0.028099
No	191 to 373	0.980942	0.019058
No	>= 373	0.079372	0.920628

Table S5D. Conditional probability table for node wind speed ($m^{-1}s^{-1}$)

Flight	Maximum temperature (C)	Outcome	
		< 4.2	>= 4.2
Yes	< 12.6	0.888888	0.111111
Yes	>= 12.6	0.877907	0.122093
No	< 12.6	0.831106	0.168894
No	>= 12.6	0.657473	0.342527

Table S5E. Conditional probability table for node maximum temperature (°C)

Flight	Photon flux density (μmol photons $m^{-2}s^{-1}$)	Outcome	
		< 12.6	>= 12.6
Yes	< 0.23	0.1	0.9
Yes	>= 0.23	1.00E-06	0.999999
No	< 0.23	0.593955	0.406045
No	>= 0.23	0.287556	0.712444

Table S5F. Conditional probability table for node Day of year

Flight	Maximum temperature (°C)	Outcome			
		< 14	14 to 41	41 to 325	>= 325
Yes	< 12.6	5.56E-07	0.444444	5.56E-07	0.555555
Yes	>= 12.6	0.040698	0.796511	5.81E-08	0.162791
No	< 12.6	3.71E-09	0.133259	0.44098	0.425761
No	>= 12.6	0.001179	0.489863	0.214286	0.294672

Table S5G. Conditional probability table for node temperature range (°C)

Flight	Time since sunset (mins)	Outcome	
		< 1.1	>= 1.1
Yes	< -20	0.333334	0.666666
Yes	-20 to 6	0.678571	0.321429
Yes	6 to 40	0.200001	0.799999
Yes	40 to 65	0.750000	0.250000
Yes	65 to 191	0.638298	0.361702
Yes	191 to 373	0.440000	0.560000
Yes	>= 373	0.333334	0.666666
No	< -20	0.59799	0.40201
No	-20 to 6	0.804348	0.195652
No	6 to 40	0.582192	0.417808
No	40 to 65	0.861386	0.138614
No	65 to 191	0.657851	0.342149
No	191 to 373	0.600897	0.399103
No	>= 373	0.314426	0.685574
